# Advanced Ankylosing Spondylitis With Posterior Mediastinal Mass: A Rare Radiological Confluence

**DOI:** 10.7759/cureus.89510

**Published:** 2025-08-06

**Authors:** Satwik Gottimukkala, Mohini Singh, Sudha Madhavan, Ramya Venkatesan, Ramakrishnan S

**Affiliations:** 1 General Medicine, Sri Ramachandra Institute of Higher Education and Research, Chennai, IND

**Keywords:** ankylosing spondylitis, lymphoma, posterior mediastinal mass, spinal cord compression, tuberculosis

## Abstract

Ankylosing spondylitis (AS) is a chronic inflammatory spondyloarthropathy that predominantly affects the axial skeleton. While hallmark features such as sacroiliitis and syndesmophytes are well recognized, the presence of mediastinal masses may pose a diagnostic dilemma and raise concerns for malignancy or atypical infection. We report a middle-aged man in the fifth decade of life with longstanding untreated AS presenting with progressive quadriparesis. Imaging revealed classical findings of bamboo spine, Romanus lesions, and sacroiliitis, alongside a mediastinal mass on chest radiograph and magnetic resonance imaging. The differential included primary malignancy, granulomatous disease, and lymphoma. Evaluation is planned further with positron emission tomography-computed tomography. This case illustrates the importance of considering extramusculoskeletal manifestations in AS, particularly mediastinal lesions that may mimic malignancy. Radiological evaluation and early intervention are essential for timely diagnosis and management.

## Introduction

Ankylosing spondylitis (AS) is a chronic seronegative arthritis that prominently involves the sacroiliac joints and vertebral column. In advanced stages, its imaging profile may resemble trauma-related deformities or spinal malignancies. Structural instability and spinal fractures, particularly in the cervical region, may lead to neurological deficits. We report a case of advanced AS with classic radiological findings complicated by cervical vertebral collapse and quadriparesis. The condition is most commonly seen in men under 40 years of age and is strongly associated with HLA-B27 positivity. Diagnosis is often delayed due to the insidious onset and overlap with mechanical back pain. Radiological imaging remains central to identifying characteristic changes such as sacroiliitis, syndesmophytes, and the bamboo spine appearance. Recent literature suggests that extramusculoskeletal manifestations, including cardiac, pulmonary, and rarely oncologic processes, may complicate the natural course of AS. This case exemplifies a complex presentation involving classical skeletal findings and an unusual mediastinal mass.

## Case presentation

A middle-aged man in his 40s with no known prior comorbidities presented with a two-week history of progressive neck pain and weakness of all four limbs. The symptoms began insidiously and included an increasing restriction of neck movements over the past three decades, which the patient had neglected. He experienced difficulty rising from bed and walking unassisted and had recurrent falls. There was associated numbness in both lower limbs extending up to the knees. The patient denied bowel or bladder incontinence, fever, or recent weight loss.

On examination, the patient was conscious and oriented, with stable vital signs. Neurological examination revealed that motor strength was reduced, suggestive of quadriparesis; hyperreflexia was present with equivocal plantar responses. There was a reduction in pain and touch sensation over both limbs below the elbow and knee levels. Cerebellar signs were absent. Gait assessment was not feasible due to marked weakness. Investigations done are enclosed in Table 1.

**Table 1:** Investigations ESR: erythrocyte sedimentation rate; CRP: C-reactive protein

Investigation	Patient value	Normal range
Hemoglobin	14.9 g/dL	13.5-17.5 g/dL
ESR	58 mm/hr	0-20 mm/hr
CRP	3.8 mg/L	<5 mg/L
Serum potassium	2.9 mmol/L (corrected to 5.0 mmol/L)	3.5-5.0 mmol/L
Vitamin D	7.29 ng/mL	20-50 ng/mL
HLA-B27	Positive	-

**Figure 1:**
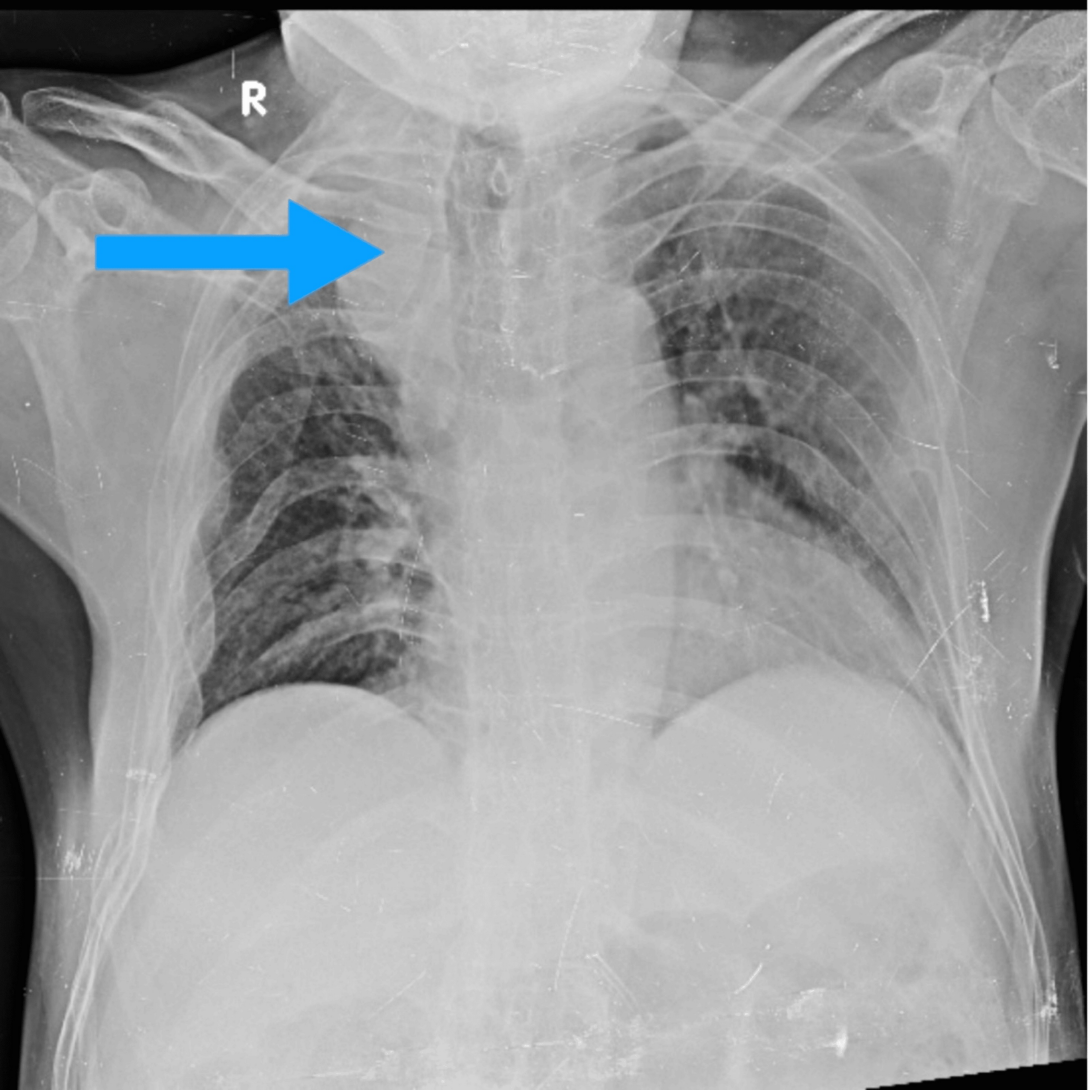
AP chest radiograph showing increased upper thoracic kyphosis. There is a probable right paratracheal opacity mass with tracheal deviation The blue arrow shows the opacity which might indicate the mass. AP: anteroposterior

**Figure 2:**
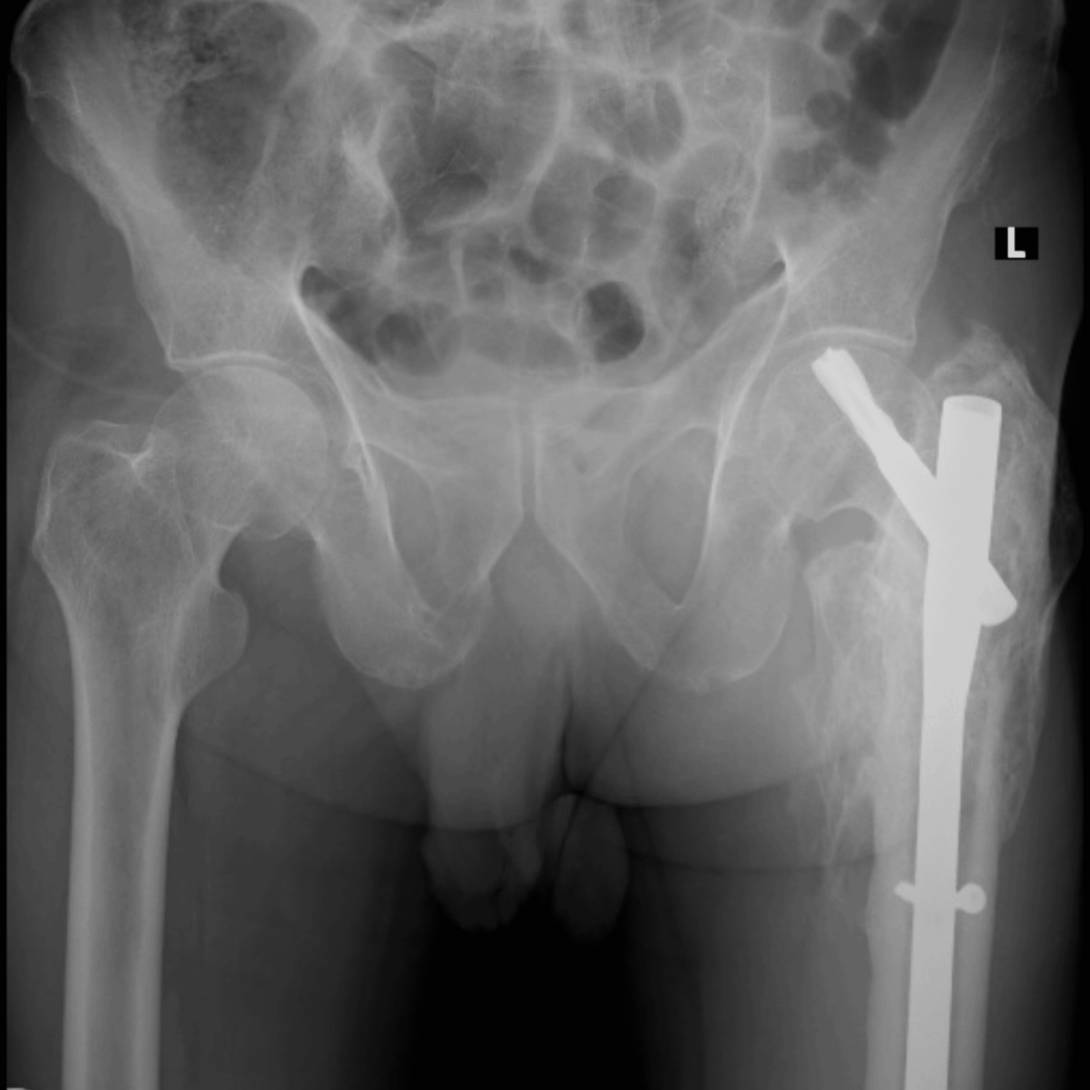
Pelvic radiograph (AP view) showing bilateral sacroiliac joint ankylosis with evidence of left-sided hip arthroplasty. The findings are typical of advanced ankylosing spondylitis with involvement of axial and appendicular skeleton AP: anteroposterior

Cervical spine radiograph showed reversal of cervical lordosis and anterior wedge compression of C6 (sixth cervical) and C7 (seventh cervical) vertebral bodies. Notably, the chest radiograph (Figure 1) revealed a right-sided paratracheal opacity with tracheal deviation, suspicious for a posterior mediastinal mass. A plain radiograph of the pelvis (Figure 2) revealed bilateral sacroiliitis with joint space narrowing and sclerosis, consistent with radiographic criteria for AS.

**Figure 3:**
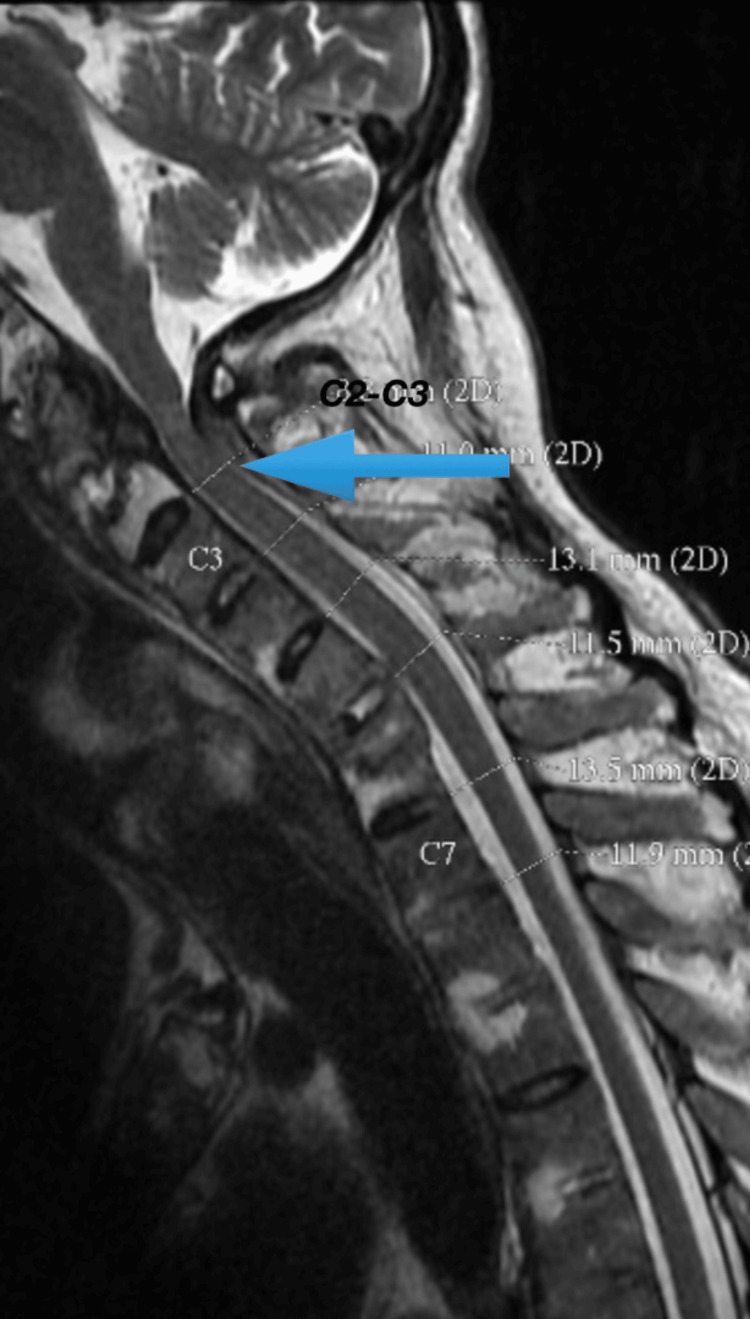
Sagittal T2-weighted MRI of the cervical spine depicting syndesmophytes and ossification of the anterior longitudinal ligament with significant spinal canal narrowing at C2-C3 (8.8 mm), raising concern for cervical canal stenosis in a patient with ankylosing spondylitis The blue arrow shows the changes of ankylosing spondylitis which is causing cord compression at the level. MRI: magnetic resonance imaging; C2: second cervical vertebra; C3: third cervical vertebra

**Figure 4:**
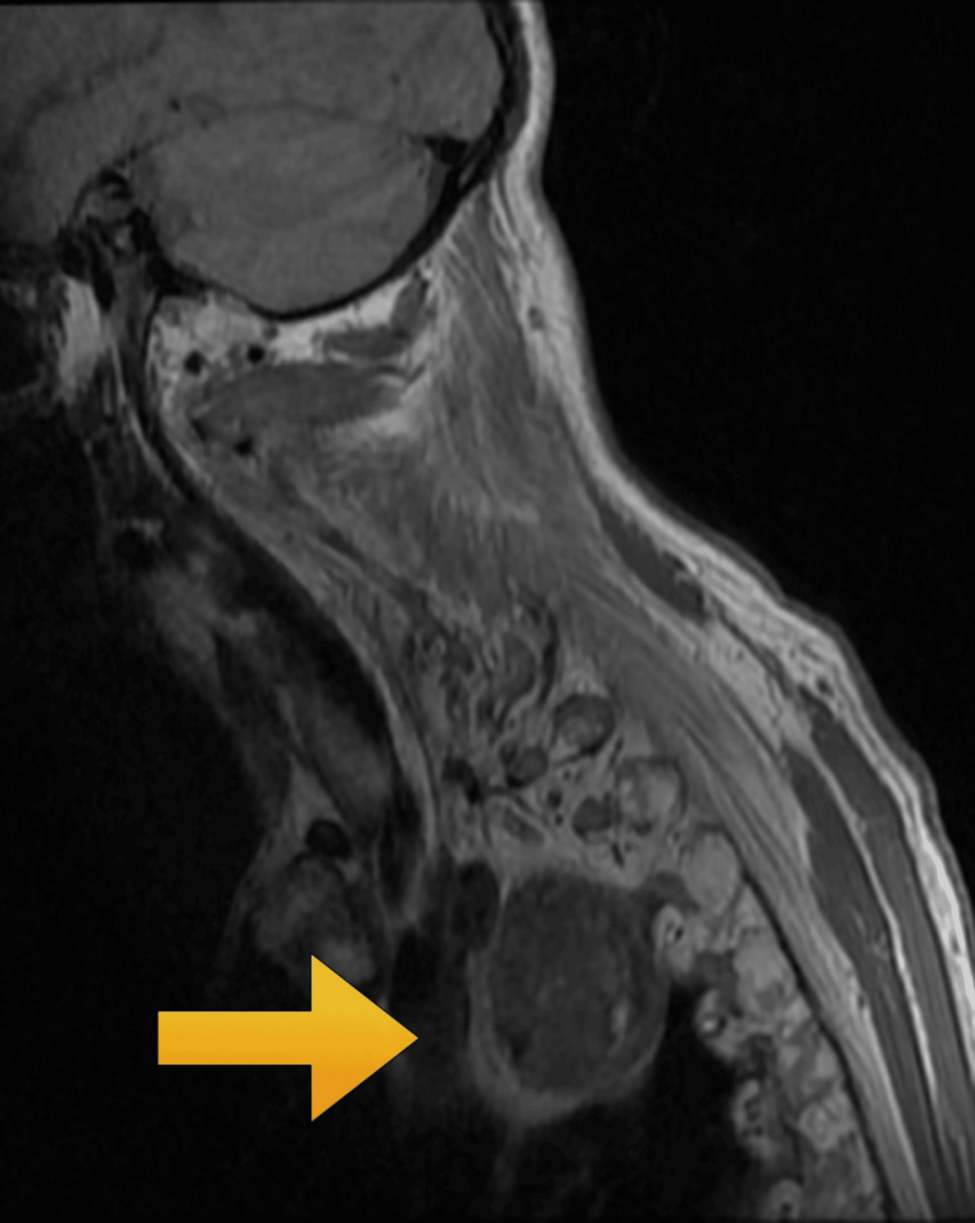
Sagittal T2-weighted MRI of the cervicothoracic junction demonstrating diffuse ossification of the anterior longitudinal ligament and loss of normal cervical lordosis, consistent with ankylosing spondylitis. Additionally, there is a well-defined, T2 hyperintense mass lesion in the posterior mediastinum, located anterior to the upper thoracic vertebrae and extending from approximately C7 to T3 vertebral levels. The lesion appears to displace adjacent soft tissue structures without frank invasion The yellow arrow shows the posterior mediastinal mass in the MRI. MRI: magnetic resonance imaging; C7: seventh cervical vertebra; T3: third thoracic vertebra

Magnetic resonance imaging (MRI) of the spine revealed a C2 (second cervical) vertebral disruption along with signal abnormalities within the spinal cord, indicative of chronic cord injury and local deformity (Figure 3). Additionally, a right posterior mediastinal lesion measuring 3.3×3.0×3.9 cm was identified (Figure 4). Whole spine MRI confirmed the classical features of AS, including syndesmophyte formation and ossified ligaments.

In our patient, MRI described a right posterior mediastinal lesion abutting vital structures (Figure 4). Differential diagnoses included (1) lymphoma, (2) tuberculosis, (3) sarcoidosis, and (4) neurogenic tumors or metastases.

The mediastinal lesion was located in the right posterior mediastinum, abutting the trachea and vertebrae, without clear evidence of neural foraminal involvement. Given the concern for probable disseminated malignancy of unknown primary origin, further evaluation with positron emission tomography-computed tomography (PET-CT) and biopsy was advised. Emergency spinal stabilization surgery was strongly recommended due to the high risk of further neurological compromise. The patient was advised to maintain strict immobilization and prescribed a cervical collar. Further tissue diagnosis via biopsy was advised; however, the patient's reluctance to proceed limited definitive categorization.

## Discussion

AS is a progressive, immune-mediated inflammatory disorder categorized under seronegative spondyloarthropathies, which preferentially affects the axial skeleton. Sacroiliitis, often symmetrical, is typically the first detectable radiological sign, progressing over time to ossification of the spinal ligaments and the intervertebral discs. This sequence results in the classic "bamboo spine" appearance on radiographs, a hallmark of advanced disease [1]. In the present case, imaging revealed hallmark features including sacroiliac joint fusion and extensive marginal syndesmophytes.

Structural damage in AS extends beyond skeletal deformity. The combination of inflammation and ossification results in significant spinal rigidity, making the spine vulnerable to fractures even with minor trauma. These fractures are typically unstable and predispose to spinal cord injuries, especially in the cervical region. Poddubnyy et al. reported that 10-30% of patients with advanced AS show radiographic progression within two years, with many developing canal stenosis and neurological deficits such as myelopathy [2]. Our case mirrored these findings, with MRI revealing significant canal narrowing and signal changes at the C2-C3 level, indicating compressive myelopathy.

Genetically, AS is strongly associated with HLA-B27 positivity, seen in more than 90% of affected individuals, suggesting a strong immunogenetic predisposition. Reveille described the potential mechanisms involving antigen misfolding, aberrant peptide presentation, and gut microbiome interaction, all contributing to chronic systemic inflammation [3]. Interestingly, emerging genetic studies suggest shared etiological links between autoimmune conditions and hematologic malignancies. A genome-wide association study by Li et al. demonstrated overlapping loci between AS and lymphoproliferative disorders, highlighting the need to consider hematological malignancy as a potential, though rare, comorbidity [4].

One of the unusual findings in our case was the detection of a posterior mediastinal mass. In the context of AS, such findings should prompt a thorough evaluation to rule out lymphoma, especially primary mediastinal B-cell lymphoma or Hodgkin lymphoma. Even in the absence of immunosuppressive therapy, there exists a risk of hematological malignancy. Mole et al. reported a case of Hodgkin lymphoma in a patient with AS post-anti-tumor necrosis factor (anti-TNF) therapy, reinforcing the importance of clinical suspicion in similar scenarios [5].

Tuberculosis remains a critical differential in endemic regions. Mediastinal tuberculosis can mimic neoplastic conditions radiologically, particularly when necrotic lymphadenopathy appears fluorodeoxyglucose (FDG)-avid on PET-CT. Martelli et al. emphasized the diagnostic challenge of distinguishing between infectious and malignant etiologies using imaging alone and underlined the role of PET-CT in lesion characterization and biopsy planning [6]. In our patient, although PET imaging was advised, tissue diagnosis could not be obtained due to the patient's refusal, which left the nature of the mass inconclusive.

MRI played a central role in assessing both spinal and mediastinal pathology. In our case, MRI revealed T2 hyperintensity of the spinal cord suggestive of compressive myelopathy as well as a posterior mediastinal mass extending from C7 to T3, which lacked definitive radiologic features to distinguish between infectious and neoplastic pathology. Absence of biopsy limited diagnostic certainty, underscoring the importance of patient counseling and shared decision-making.

Beyond skeletal involvement, AS can affect multiple organ systems. Cardiovascular manifestations, such as aortic root dilation, valvular regurgitation, and conduction system abnormalities, and pulmonary features, like apical fibrosis, are documented extra-articular manifestations [7]. While absent in this case, their presence necessitates a systemic approach in AS management. Hematological abnormalities, although less frequently reported, are emerging in recent literature, especially in chronic inflammatory states or in association with biological therapies [4,5].

Management of AS complicated by neurologic compromise and incidental mediastinal pathology must be multidisciplinary. In our case, input from internal medicine, neurology, spine surgery, radiology, rheumatology, and oncology was essential in formulating a comprehensive plan. Surgical decompression was prioritized due to cord compression. The 2016 Assessment of Spondyloarthritis International Society (ASAS)-European Alliance of Associations for Rheumatology (EULAR) update reiterates individualized management strategies, especially in advanced disease stages or with extra-skeletal complications [8].

This case reinforces the need for heightened clinical vigilance. In AS patients presenting with atypical features like a mediastinal mass, differential diagnoses must include lymphoma, tuberculosis, and sarcoidosis. Radiologic tools such as MRI and PET-CT, while invaluable, cannot replace tissue diagnosis, which remains the gold standard. Clinicians must maintain a broad differential and be prepared for rare yet significant complications [6,9].

The role of integrated imaging modalities has expanded. Newer approaches, such as whole-body MRI and PET-MRI fusion, are being explored in chronic inflammatory disorders like AS, offering both structural and metabolic information in a single scan. These could bridge current diagnostic gaps, particularly when patients decline biopsy or when lesions are inaccessible [10]. Additionally, recent reviews have suggested that long-standing AS is an independent risk factor for malignancies, particularly lymphomas and gastrointestinal (GI) tract cancers, emphasizing the need for long-term surveillance [11].

Differential diagnosis for posterior mediastinal mass in AS is discussed in Table 2.

**Table 2:** Differential diagnosis for posterior mediastinal mass in AS PMBCL: primary mediastinal B-cell lymphoma; FDG: fluorodeoxyglucose; PET-CT: positron emission tomography-computed tomography; AS: ankylosing spondylitis; TNF-α: tumor necrosis factor-alpha; TB: tuberculosis; MRI: magnetic resonance imaging

Diagnosis	Evidence for	Evidence against	References
Lymphoma (e.g., Hodgkin, PMBCL)	FDG-avid lesion on PET-CT suggestive of high metabolic activity [6]. Common cause of mediastinal mass in adults. Known risk in AS, especially with TNF-α therapy [5]	No B-symptoms reported. No lymphadenopathy elsewhere. No biopsy confirmation due to patient refusal	[5,6,11]
TB (mediastinal TB)	High prevalence in India. Can present with necrotic lymph nodes and mimic malignancy on PET-CT [6]. Systemic inflammation may predispose to reactivation	No systemic symptoms (fever, weight loss, night sweats). No sputum positivity or evidence of pulmonary TB. No tissue diagnosis	[6,11]
Neurogenic tumor (e.g., schwannoma)	Most common cause of posterior mediastinal mass. Well-defined and T2 hyperintense on MRI, as in this case [10]	Age less typical (more common in younger adults). No mention of "dumbbell shape" or neural foraminal extension. No enhancement pattern seen	[10]
Sarcoidosis	Can involve the mediastinum and mimic lymphoma. May cause hilar and mediastinal lymphadenopathy	No lung parenchymal involvement. No hypercalcemia or skin/eye findings. Rarely isolated mediastinal mass in the absence of other systemic findings	[9]
Metastasis (occult primary)	Mass effect in the posterior mediastinum may indicate metastatic spread	No known primary tumor. No other metastases found. Imaging lacked aggressive features	[9,11]
IgG4-related disease	Known to cause fibroinflammatory mediastinal lesions. Chronic systemic inflammation like AS may trigger autoimmunity	No biopsy; hence, no IgG4-positive plasma cells identified. No typical other organ involvement (e.g., pancreas, salivary glands)	[10,11]
Paraspinal abscess (infective)	Possible in immunocompromised or chronic inflammatory state. Can appear as soft tissue mass	No fever or signs of systemic infection. Imaging did not reveal abscess characteristics (no rim enhancement or diffusion restriction)	[6,11]
Bronchogenic cyst/developmental cyst	T2 hyperintense well-circumscribed lesion on MRI may support cystic lesion	No fluid-fluid level or classical MRI appearance of simple cyst. Less common in adults. Location more typical in the middle/posterior mediastinum	[10]

## Conclusions

This case highlights the complex and often under-recognized interplay between advanced AS and non-musculoskeletal complications, such as mediastinal masses. It underscores the diagnostic challenges faced when radiological findings overlap with potential malignancies or infections. Timely and comprehensive imaging is essential not only for identifying classical features like bamboo spine and sacroiliitis but also for detecting potentially life-threatening complications, such as cervical instability or space-occupying lesions. Neurological deficits, especially quadriparesis, demand urgent evaluation and surgical intervention to prevent irreversible damage. Additionally, the incidental detection of a mediastinal mass in a patient with chronic inflammatory disease necessitates a broad differential diagnosis and a multidisciplinary approach. Ultimately, this case reaffirms the need for early diagnosis, regular monitoring, and collaborative management strategies in patients with advanced spondyloarthropathies.
